# The therapeutic potential of vitamins A, C, and D in pancreatic cancer

**DOI:** 10.1016/j.heliyon.2024.e41598

**Published:** 2024-12-31

**Authors:** Alban Piotrowsky, Markus Burkard, Hendrik Schmieder, Sascha Venturelli, Olga Renner, Luigi Marongiu

**Affiliations:** a Department of Nutritional Biochemistry, University of Hohenheim, 70599, Stuttgart, Germany; b Institute of Physiology, Department of Vegetative and Clinical Physiology, University Hospital Tuebingen, 72076, Tuebingen, Germany; c Faculty of Food and Nutrition Sciences, University of Applied Sciences, Hochschule Niederrhein, 41065, Moenchengladbach, Germany; d HoLMiR-Hohenheim Center for Livestock Microbiome Research, University of Hohenheim, 70599, Stuttgart, Germany

**Keywords:** Vitamins, Cancer, Pancreatic cancer, Ascorbic acid, Calcitriol, ATRA

## Abstract

The pancreatic ductal adenocarcinoma (PDAC) is among the deadliest tumor diseases worldwide. While treatment options have generally become more diverse, little progress has been made in the treatment of PDAC and the median survival time for patients with locally advanced PDAC is between 8.7 and 13.7 months despite treatment. The aim of this review was to explore the therapeutic potential of complementing standard therapy with natural or synthetic forms of vitamins A, C, and D.

The therapeutic use of vitamins A, C, and D could be a promising addition to the treatment of PDAC. For all three vitamins and their derivatives, tumor cell-specific cytotoxicity and growth inhibition against PDAC cells has been demonstrated in vitro and in preclinical animal models. While the antitumor effect of vitamin C is probably mainly due to its pro-oxidative effect in supraphysiological concentrations, vitamin A and vitamin D exert their effect by activating nuclear receptors and influencing gene transcription. In addition, there is increasing evidence that vitamin A and vitamin D influence the tumor stroma, making the tumor tissue more accessible to other therapeutic agents.

Based on these promising findings, there is a high urgency to investigate vitamins A, C, and D in a clinical context as a supplement to standard therapy in PDAC. Further studies are needed to better understand the exact mechanism of action of the individual compounds and to develop the best possible treatment regimen. This could contribute to the long-awaited progress in the treatment of this highly lethal tumor entity.

## Abbreviations

1,25(OH)_2_D, or calcitriol: 1,25-dihydroxyvitamin D
25(OH)D: 25-hydroxyvitamin D
5-FU: 5-fluorouracil
ATRA: all-trans-retinoic acid
AQP: aquaporins
BAX: Bcl-2-like protein 4
Bcl-2: B-cell lymphoma 2
CRABP: cellular retinoic acid binding protein
Chmp1A: charged multivesicular body protein 1A
DNMT: DNA methyltransferase
EGFR: epidermal growth factor receptor
EMT: epithelial-mesenchymal transition
EPIC: European Prospective Investigation into Cancer and Nutrition
GEM: gemcitabine
HIF-1α: hypoxia inducible factor 1 subunit alpha
JNK: c-Jun N-terminal kinase
MLC-2: myosin light chain 2
MMP-1: matrix metalloproteinase-1
nabP: nab-paclitaxel
OS: overall survival
PAK: p21-activated kinases
PDAC: pancreatic ductal adenocarcinoma
PSC: pancreatic stellate cells
ROS: reactive oxygen species
RAR: retinoic acid receptors
RXR: retinoid X receptor
VEGF: vascular endothelial growth factor
VDBP: vitamin D binding protein
VDR: vitamin D receptor

## 1 Introduction

Pancreatic malignant neoplasms are currently the tenth most common newly diagnosed cancer with 66,440 estimated cases emerging in 2024 in the United States. However, with 51,750 estimated deaths, pancreatic tumors are the third leading cause of tumor-associated deaths [1], and are expected to move up to second place by 2040 [2]. An increasing incidence of this tumor disease is observed for both men and women, with a significant increase in new cases between 2017 and 2021 in the United States [3]. Pancreatic ductal adenocarcinoma (PDAC) is one of the most aggressive tumors with poor prognosis and a current 5-year survival rate of 13 % for all stages combined and only 3 % for patients with distant metastatic disease stage. Even in the small percentage of patients with localized disease at diagnosis, the 5-year survival rate is only 44 % [4]. While a decrease in mortality has been observed for most malignancies within recent years due to advances in therapy, the mortality rate for PDAC in the U.S. remained unchanged in women over the 2016–2020 period and actually increased slightly in men [3].

Cancer therapy in general has developed a great deal. While initially only surgical removal, radiation, and cytotoxic agents were available, which mainly affected rapidly dividing cells, tamoxifen was the first important non-cytotoxic small molecule to find its way into cancer therapy [5]. This was followed by numerous molecularly targeted cancer treatments. The growing understanding of oncogenic somatic and germline mutations in oncogenes and tumor suppressor genes led to the development of further therapeutic approaches and today allogeneic hematopoietic stem cell transplantation, hormone therapy, therapies targeting oncogenic alterations, antibody drug conjugates, immune checkpoint inhibitors, bispecific T-cell engagers, oncolytic virotherapy, chimeric antigen receptor T-cell therapy, cancer vaccines, microRNA, pulsed electromagnetic field therapy, and numerous others are available [5].

In general, the prognosis of PDAC as well as the initiative treatment for patients with local or regional disease, depends on the resectability of the tumor at the time of diagnosis [6]. To date, the only chance of cure for pancreatic cancer is a combination of complete resection and systemic multi-agent chemotherapy [7]. The decision whether surgical resection of the tumor is feasible depends on the stage and exact location of the neoplasm, for instance contact with large blood vessels, as well as the age and general health status of the patient [8]. Tumors distal to the pancreatic head are usually attempted to be removed by distal pancreatectomy. However, the majority of pancreatic tumors are located in the pancreatic head and require pancreaticoduodenectomy, also known as the Whipple procedure [9]. Because the recurrence rate continues to be very high at 70–80 %, adjuvant chemotherapy follows resection as standard [10]. In patients in whom surgery is not possible or appropriate, chemotherapy may be given to prolong life or to improve quality of life [11]. In addition, in patients with borderline resectable tumors and locally advanced disease, neoadjuvant chemotherapeutic treatment may be useful [12]. Whereas, for many years, gemcitabine and 5-fluorouracil were established as standard chemotherapeutic agents, clinical trials have now demonstrated the superiority of both FOLFIRINOX (combination of leucovorin, fluorouracil, irinotecan, and oxaliplatin) and gemcitabine combined with nab-paclitaxel (nabP) over gemcitabine monotherapy [13,14]. Here, depending on stage of disease at diagnosis as well as patient performance status, the recommendation for choice of adjuvant chemotherapy differs. While FOLFIRINOX is predominantly used in patients with good physical condition, gemcitabine-nabP is chosen as standard therapy in patients with low performance status, concomitant disease, and advanced age [6]. This is based on the findings that FOLFIRINOX prolongs patients' overall survival (OS) compared to gemcitabine with nabP, but is associated with significantly more severe side effects [15].

The poor prognosis of this disease is due to early systemic spread and aggressive local growth [6]. Further complicating the prognosis, approximately 50–60 % of patients are at the distant metastatic disease stage at diagnosis, 25–30 % are at the regional disease stage, and only 10–15 % of patients are at the local disease stage at diagnosis [16]. The advanced stage at the time of diagnosis is primarily due to the initial mild and nonspecific symptoms, usually only becoming more persistent and specific as the disease progresses [17]. These may include weight loss, abdominal pain radiating to the back, jaundice, vomiting, and digestive problems [4,18]. Furthermore, the therapeutic success of current standard chemotherapeutic agents is limited, only minor therapeutic advances have been achieved within the past years and decades, and new therapeutic approaches such as immune checkpoint inhibitors, which have achieved success in other tumor entities, have been predominantly unsuccessful in PDAC [19]. The poor prognosis of this disease is also due to rapid metastasis, aggressive growth, dense tumor stroma, and inadequate treatment options [6]. Consequently, despite the use of current standard therapies for patients with locally advanced pancreatic cancer, a median OS of between 8.7 and 13.7 months is achieved [20]. The persistent poor prognosis for patients with pancreatic cancer of all stages, the significant side effects of existing treatment options, and the limited success of most newer treatment approaches underscore the urgency of exploring new treatment options, as well as palliative approaches, to improve patients' quality of life.

In this context, this review focuses on the latest findings on three promising micronutrients as additional therapeutic approaches for the treatment of PDAC, namely vitamin A, vitamin C, and vitamin D. For all three vitamins, a high prevalence of deficiencies was found in patients with various tumor entities, including PDAC [21,22] and the immune environment of cancers has become an increasingly important topic [23,24]. In addition, deficiencies of these vitamins are associated with lower median survival in PDAC patients, raising great interest in the use of these natural substances in tumor therapy [[25], [26], [27]].

## 2 Complementary therapeutic approaches in pancreatic cancer

### 2.1 Vitamin A

#### 2.1.1 Metabolism and function

**Fig. 1:**
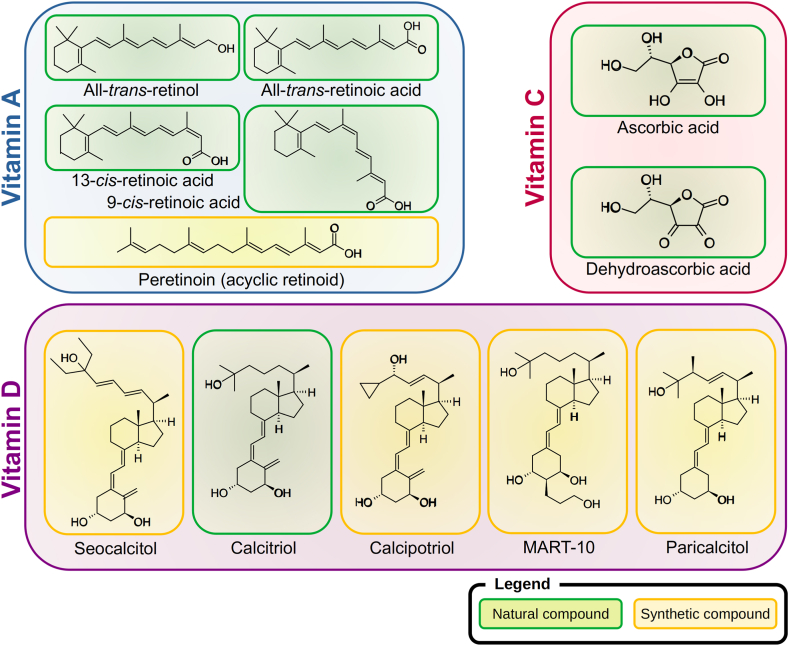
Chemical structures of relevant natural and synthetic forms of vitamins A, C, and D. Created with ACD/ChemSketch software 2022.2.0, Advanced Chemistry Development, Inc., Toronto, Canada.

The chemical structures of relevant natural and synthetic forms of vitamins A, C, and D are shown in Fig. 1. Vitamin A is an essential micronutrient for the human organism. The term “vitamin A″ includes compounds that exhibit the biological activity of retinol. In addition to retinol, this includes retinyl esters, retinal, retinoic acid, and oxidized as well as conjugated forms of both retinol and retinal. The term “retinoids” includes naturally occurring forms of vitamin A as well as synthetic retinol analogues [28]. Vitamin A performs different important functions in the human organism. One of the most well-known functions is its involvement in the visual process, with vitamin A acting as a precursor of 11-cis-retinal, which in combination with the protein opsin forms the complex called rhodopsin, being the light-sensitive pigment in the rods of the eyes [29,30]. Furthermore, vitamin A fulfills important functions in the regulation of both growth and differentiation of cells, as well as immune cell function and bone and tooth growth [28].

Dietary vitamin A is absorbed either in the form of retinyl esters (preformed vitamin A) or as carotenoids (provitamin A) [31,32]. Vitamin A that is not immediately needed in the organism is stored esterified in liver stellate cells, making the liver the main storage organ for this molecule [32]. When vitamin A is needed, it is released from storage by hydrolysis of the ester bond and retinol is coupled to the specific transport protein, retinol binding protein, whereupon this complex can enter the systemic circulation and be taken up by peripheral cells [32]. In target cells, after uptake, retinol is converted to all-trans-retinal and bound to cellular retinol binding protein, resulting in enzymatic oxidation to the major biologically active metabolite, all-trans-retinoic acid (ATRA), which in turn is bound by cellular retinoic acid binding protein (CRABP) [28].

The action of vitamin A is mediated mainly by retinoic acid receptors (RAR) and retinoid X receptors (RXR). Each of these receptor families contain three isoforms (α, β, and γ) [33]. After ligand binding, they serve as transcription factors [28]. RXR activation is mediated by 9-cis-retinoic acid while RAR activation is induced by 9-cis-retinoic acid and ATRA. The latter mediates the majority of cellular vitamin A signaling [34]. Furthermore, ATRA has also been shown to act as a ligand for peroxisome proliferator-activated receptors [35].

The activation and repression of gene transcription by binding of ATRA to various nuclear receptors describes the genomic effects of retinoids. In addition, however, retinoids can exert nongenomic effects or non-RAR/RXR-mediated nonclassical effects, some of them much more short-term. For example, both ATRA and retinol induce phosphorylation of the cAMP response element binding protein, which in turn results in the activation of genes containing the cAMP response element in their promoter region [36,37]. Furthermore, there is evidence that RARα can act directly in the cytoplasm as an RNA binding protein after activation, inhibiting translation of certain proteins [38].

#### 2.1.2 Vitamin A and pancreatic cancer risk

To date, several studies have been conducted to evaluate a possible association between vitamin A intake and the risk of developing a pancreatic tumor. Overall, these studies have provided mixed, sometimes contradictory, conclusions. An early systematic review by Bjelakovic et al. showed no association between vitamin A intake and risk of pancreatic cancer [39]. A case control study performed by Zablotska et al., failed to find a statistically significant association between retinol intake and pancreatic cancer risk for both men and women [40]. In the nested case control study European Prospective Investigation into Cancer and Nutrition (EPIC), no association was found either [41]. In contrast, two meta-analyses of 11 epidemiological studies showed an inverse association between dietary vitamin A intake and risk of disease for pancreatic cancer [42]. The most recent meta-analysis by Zhang et al. also found evidence of a possible negative association between dietary vitamin A intake and pancreatic cancer incidence [43]. Overall, no clear statement on the association between vitamin A intake and pancreatic cancer incidence can be made at present, but a causal relationship seems unlikely due to the very heterogeneous, partly contradictory study situation.

#### 2.1.3 Vitamin A and pancreatic cancer

For over 20 years, ATRA has been used as a standard chemotherapeutic agent for the treatment of certain forms of leukemia, for example acute promyelocytic leukemia [44,45]. Interest in the role of vitamin A in tumor therapy and specifically PDAC therapy is reinforced by the finding that, although human pancreatic cancer cell lines express the nuclear receptors RARα, RARβ, RXRα, and RXRβ necessary for vitamin A action [46,47], these are often downregulated in PDAC tissue compared to healthy tissue [48]. Furthermore, this decreased expression of nuclear receptors has been shown to correlate with the expression of various markers of cell differentiation and epithelial-mesenchymal transition (EMT) as well as tumor stem cell markers. Moreover, the expression of RARα and RXRβ is associated with higher OS in pancreatic cancer patients. Furthermore, ATRA and all-trans-retinol levels have been shown to be reduced in PDAC tissue compared to healthy pancreatic tissue [48].

**Fig. 2:**
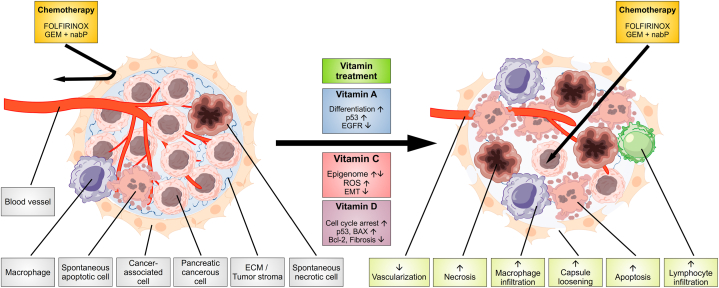
Therapeutic potential and target points of vitamins A, C, and D in the treatment of pancreatic cancer. Conventional therapeutic approaches are largely ineffective in pancreatic cancer, as a dense tumor stroma consisting of PSC, cancer-associated fibroblasts and ECM makes tumor accessibility by therapeutics difficult and the tumor cells show a low response to most forms of therapy. By combining conventional therapeutic approaches with various forms of vitamins A, C, or D, an improved antitumor effect could be achieved. The effect of vitamin A is induced by increased differentiation of tumor cells, re-expression of p53 and EGFR downregulation. Vitamin C exerts its antitumor effect through ROS induction, epigenome regulation, and EMT inhibition. Vitamin D and its analogues reduce fibrosis in the tumor stroma, lead to senescence of PSC and inhibit tumor growth by inducing cell cycle arrest and apoptosis. Together, the vitamins reduce proangiogenic signals. BAX: Bcl-2-like protein 4; Bcl-2: B-cell lymphoma 2; ECM: extracellular matrix; EGFR: eucaryotic growth factor receptor; EMT: epithelial-mesenchymal transition; GEM: gemcitabine; nabP: nab-paclitaxel; PSC: pancreatic stellate cells; ROS: reactive oxygen species.

The potential effects of vitamin treatment in PDAC, in addition to standard therapy, are diverse (Fig. 2). For various forms of vitamin A, antitumor effects have been found in vitro at supraphysiological concentrations in a variety of cancer cell lines of different tumor entities, including pancreatic cancer (Table 1) [49,50]. In addition to 9-cis-retinoic acid and 13-cis-retinoic acid, this applies in particular to ATRA [46,47]. It has been shown several times that ATRA inhibits tumor cell growth and induces cytotoxicity via various effects. These include ATRA-mediated reduced proliferation, colony formation, migration, and invasion, in both wild-type and gemcitabine-resistant human pancreatic cancer cell lines [51]. Of note, however, there is also a single study that also demonstrated an inhibitory proliferation effect of ATRA against a human pancreatic cancer cell line, but with concomitantly increased invasive potential of the cells [52].

**Table 1:** Effects of natural and synthetic isoforms of vitamins A, C, and D on PDAC cells in vitro and in vivo.

Compound	Derivative/Analogue	Effect	Mechanism	References
Vitamin A	All-trans retinoic acid	Proliferation ↓, migration ↓, colony formation ↓, invasion ↓, PSC quiescence ↑, PSC motility ↓, apoptosis ↑, tumor growth in mice ↓, synergism with GEM and nabP	PAK expression ↓, MLC-2 expression ↓, differentiation ↑, p53 expression ↑, EGFR expression ↓, HsEg5 expression ↓	[51,54,56,57,[68], [69], [70],^73^]
	9-cis-retinoic acid	Proliferation ↓, apoptosis ↑, synergism with cisplatin and GEM	p27 expression ↑, Bcl-2/Bax ratio ↓	[50,[76], [77], [78]]
	13-cis-retinoic acid	Proliferation ↓	Regulation of MMP-1 activity, JNK pathway activity ↓	[46,75,79]
	Peretinoin (acyclic retinoid)	Tumor growth in mice ↓	–	[62]
Vitamin C	Ascorbic acid	Proliferation ↓, EMT ↓, metastasis ↓, synergism with GEM, nabP, 5-FU, FOLFIRINOX, DNMT inhibitors, immunotherapy, and radiotherapy, tumor growth in mice ↓, good tolerability, and indications of antitumor activity in clinical trials	ROS ↑, kinase activity ↓, HIF-1α activity ↓, VEGF expression ↓, ATP ↓, Wnt/β-catenin signaling ↓	[[80], [81], [82], [83], [84], [85], [86], [87], [88], [89], [90], [91], [92]]
Vitamin D	Calcitriol	Proliferation ↓, cellular gemcitabine uptake ↑, synergism with GEM	p21 expression ↑, p27 expression ↑, hedgehog pathway ↓, expression of efflux proteins ↓	[[93], [94], [95], [96], [97], [98], [99]]
	MART-10	EMT ↓	–	[100]
	Calcipotriol	Proliferation ↓, PSC quiescence ↑, stroma fibrosis in mice ↓, tumor growth in mice ↓, synergism with GEM and oncolytic viroimmunotherapy	Wnt/β-catenin signaling ↓	[95,96,[101], [102], [103]]
	Seocalcitol	Proliferation ↓, tumor growth in mice ↓, good tolerability in a clinical trial	–	[[104], [105], [106], [107]]
	Paricalcitol	Proliferation ↓, synergism with GEM in mice, good tolerability in a clinical trial	p21 expression ↑, p27 expression ↑	[99,108,109]

5-FU: 5-fluorouracil; BAX: Bcl-2-like protein 4; Bcl-2: B-cell lymphoma 2; DNMT: DNA methyltransferase; EGFR: epidermal growth factor receptor; EMT: epithelial-mesenchymal transition; GEM: gemcitabine; HIF-1α: hypoxia inducible factor 1 subunit alpha; JNK: c-Jun N-terminal kinase; MLC-2: myosin light chain 2; MMP-1: matrix metalloproteinase-1: nabP: nab-paclitaxel; PAK: p21-activated kinases; PDAC: pancreatic ductal adenocarcinoma; PSC: pancreatic stellate cells; ROS: reactive oxygen species; VEGF: vascular endothelial growth factor.

A major obstacle in pancreatic cancer chemotherapy is the impaired activity of pancreatic stellate cells (PSC), which produce fibrotic stroma in the tumor environment and thus impede the delivery of drugs to the tumor [53]. Healthy PSCs are quiescent, whereas in pancreatic tumors, activation of these cells is often observed, leading to secretion and remodeling of a stiff extracellular matrix [54]. To overcome this problem, possibilities of magnetic nanoparticle based targeted drug delivery are being investigated. In this context, it was found that magnetic nanoparticles loaded with gemcitabine and ATRA can be taken up by both pancreatic cancer cells and PSC and lead to the elimination of both cell types [55]. Treatment with ATRA further restored PSC quiescence in vitro [56] and in mice, ATRA was shown to prevent cancer cell invasion by reducing PSC motility and induce apoptosis in surrounding tumor tissue [57].

In addition to the antitumor efficacy of retinoid monotherapy, a synergistic effect has been noted with some common standard chemotherapeutics for the treatment of PDAC, particularly gemcitabine. For example, ATRA in combination with gemcitabine was found to synergistically inhibit cell growth of non-gemcitabine-resistant and gemcitabine-resistant pancreatic cancer cells [51,58]. A hallmark of gemcitabine-resistant pancreatic cancer cell lines is an increased expression of p21-activated kinases (PAK), whereas PAK inhibition is associated with decreased tumor growth (up to 90 %) and increased gemcitabine sensitivity [51]. In this context, ATRA led to decreased expression of several PAKs in human pancreatic cancer cells and thus, in addition to independent cytotoxicity, was able to synergistically reduce tumor cell growth when combined with gemcitabine [51]. Preincubation of cells with ATRA reduced the half-maximal inhibitory concentration of gemcitabine by a factor of 2.8 in a gemcitabine-resistant PDAC cell line [59]. Both ATRA and 9-cis-retinoic acid were shown to have synergistic effects with cisplatin in addition to gemcitabine by preincubation of pancreatic cancer cells, and preliminary evidence for their efficacy has also been collected in animal models [50]. In addition to the numerous in vitro studies, the antitumor efficacy of retinoic acid has also been confirmed by some in vivo experiments. For example, treatment of human pancreatic carcinoid xenografts in mice with ATRA over a period of one month resulted in a dose-dependent inhibition of tumor growth up to 63 % [60]. Similar results were confirmed in further studies in vivo, where ATRA treatment (10 mg/kg/d orally) was found to reduce activated stroma, tumor cell proliferation and increase apoptosis [57,61]. A first in vivo study on the combination treatment of gemcitabine and the synthetic acyclic retinoid peretinoin in a PDAC mouse model confirmed a synergistic effect and showed a clear inhibition of tumor growth, whereas the growth of healthy pancreatic epithelial cells remained unaffected [62].

To date, only a small number of clinical studies have been conducted on vitamin A in the treatment of pancreatic cancer (Table 2). A small phase Ib study for patients with advanced, unresectable PDAC evaluated ATRA as a stromal agent in combination with standard chemotherapy consisting of gemcitabine and nabP and concluded that this combination was safe and tolerable, with evidence of stromal modulation [63]. Although this evidence needs to be confirmed in further studies, such stromal modulation could increase the tumor accessibility of current PDAC drugs and therefore lead to a synergistic effect. As a next step, the same authors plan to evaluate the efficacy of ATRA in combination with gemcitabine and nabP in pancreatic cancer in a randomized phase II trial (NCT04241276, not yet recruiting). A phase II trial evaluated a combination of 13-cis-retinoic acid with gemcitabine in patients with unresectable PDAC [64]. However, although the combination therapy was well tolerated, no improvement in response rate was observed. Another ongoing early phase I clinical trial is evaluating the efficacy of ATRA in combination with the immune checkpoint inhibitor nivolumab; results are expected in early 2025 (NCT05482851, enrollment).

**Table 2:** Completed and ongoing clinical trials of vitamins A, C, D, or their analogues in PDAC patients.

Compound	NCT Number	Phase	Disease	Age of Patients	Treatment	Enrollment	Study Status
Vitamin A	NCT03307148	1	Pancreatic Adenocarcinoma	≥18 Years	nabP, GEM, ATRA	29	Completed
	NCT04241276	2	Pancreatic Cancer	≥16 Years	nabP, GEM, ATRA	170	Not Yet Recruiting
	NCT05482451	1	Advanced or Metastatic Pancreatic Cancer	20–100 Years	Nivolumab, ATRA	20	Recruiting
Vitamin C	NCT01049880	1	Pancreatic Neoplasms	≥18 Years	GEM, Asc	15	Completed
	NCT01905150	2	Pancreatic Cancer	≥18 Years	G-FLIP, Asc	34	Completed
	NCT03410030	1, 2	Pancreatic Cancer	≥18 Years	GEM, nabP, Cisplatin, Asc	27	Completed
	NCT03146962	2	Pancreatic/Colorectal/Lung Cancer	≥18 Years	Asc (Pre-operative)	61	Completed
	NCT01364805	1	Pancreatic Cancer	≥21 Years	GEM, Asc	14	Completed
	NCT02896907	1	Pancreatic Cancer	18–75 Years	FOLFIRINOX, Asc	8	Completed
	NCT00954525	1	Metastatic Pancreatic Cancer	18–75 Years	GEM, Erlotinib, Asc	14	Completed
	NCT03541486	2	Pancreatic Neoplasms	≥18 Years	Radiation Therapy, GEM, Asc	60	Not Yet Recruiting
	NCT04033107	2	Pancreatic/Hepatocellular/Gastric/Colorectal Cancer	18–70 Years	Metformin, Asc	30	Recruiting
	NCT06018883	3	Metastatic Pancreatic Adenocarcinoma	18–80 Years	nabP, GEM, Asc	100	Recruiting
	NCT01852890	1	Pancreatic Neoplasms	≥18 Years	Radiation Therapy, GEM, Asc	16	Active, Not Recruiting
	NCT02905578	2	Pancreatic Cancer	≥18 Years	nabP, GEM, Asc	65	Active, Not Recruiting
Vitamin D	NCT03883919	1	Pancreatic Cancer	≥18 Years	5-FU, LV, Liposomal Irinotecan, Paricalcitol	20	Completed
	NCT03331562	2	Pancreatic Cancer	≥18 Years	Pembrolizumab, Paricalcitol	24	Completed
	NCT02030860	N/A	Pancreatic Adenocarcinoma	≥18 Years	Abraxane, GEM, Paricalcitol	15	Completed
	NCT02930902	1	Pancreatic Cancer	≥18 Years	GEM, nabP, Pembrolizumab, Paricalcitol	9	Completed
	NCT04524702	2	Advanced or Metastatic Pancreatic Cancer	≥18 Years	nabP, GEM, Hydroxychloroquine, Paricalcitol	21	Recruiting
	NCT05365893	1	PDAC	≥18 Years	Losartan, Hydroxychloroquine, Paricalcitol (Pre-operative)	20	Recruiting
	NCT03520790	1, 2	Pancreatic Cancer	≥18 Years	nabP, GEM, Paricalcitol	36	Active, Not Recruiting
	NCT04617067	2	Advanced Pancreatic Cancer	≥18 Years	nabP, GEM, Paricalcitol	15	Active, Not Recruiting
	NCT03415854	2	Pancreatic Cancer	≥18 Years	nabP, GEM, Cisplatin, Paricalcitol	14	Active, Not Recruiting
	NCT02754726	2	Metastatic PDAC	≥18 Years	nabP, GEM, Nivolumab, Cisplatin, Paricalcitol	10	Active, Not Recruiting

5-FU: 5-fluorouracil; Asc: ascorbic acid; ATRA: all-trans-retinoic acid; GEM: gemcitabine; G-FLIP: GEM, 5-FU, folinic acid, irinotecan, cisplatin; LV: Leucovorin; nabP: nab-paclitaxel; PDAC: pancreatic ductal adenocarcinoma.

#### 2.1.4 Antitumor mechanism of vitamin A

The main antitumor activity of retinoids is most likely due to their function as activators of the nuclear receptors RAR and RXR [65]. It was shown early on that retinoid treatment leads to growth inhibition only in pancreatic cancer cell lines expressing different forms of RAR [66]. In addition, decreased expression of retinoic acid-binding nuclear receptors appears to play a role in the pathogenesis of PDAC. Activation of RARβ transcriptionally leads to decreased expression of the protein myosin light chain 2 in pancreatic cancer cells. Since this protein is an important component of the contractile actomyosin apparatus, its downregulation leads to inhibition of tumor cell invasive capabilities [67]. Through this mechanism, ATRA also leads to mechanical quiescence in PSCs involved in tumor cell invasion [54,68]. As another mechanism, ATRA has been shown to lead to increased expression of transdifferentiation and redifferentiation markers in pancreatic cancer cells with multipotent stem cell potential in addition to increased p53 expression in these cells, indicating induction of tumor cell differentiation by ATRA [69]. Consistent with this, retinoic acid has been shown to reduce the expression of the pancreatic stem cell markers CD24, CD44, CD133, and aldehyde dehydrogenase 1 [58]. Regarding the mechanism of action, it was further found that the epidermal growth factor receptor (EGFR) expression in pancreatic cancer cells could be reduced by retinoids such as ATRA, which was reflected by inhibition of EGFR ligand-induced proliferation [69,70]. In addition, it could be shown that ATRA can increase the expression of deoxycytidine kinase in PDAC cell lines, the enzyme that catalyzes the rate-limiting step of gemcitabine activation, which could explain a synergism of both substances [59].

Furthermore, several molecular markers were identified that determine the sensitivity of human pancreatic cancer cells to retinoic acid. Interestingly, increased fatty acid binding protein 5 expression seems to be associated with decreased ATRA cytotoxicity and growth inhibition and even to increase the invasive and migratory potential of tumor cells, without knowing the underlying mechanism of action. On the other hand, increased expression of CRABP2 is associated with significantly increased ATRA inhibition [61]. In agreement with this, it has been shown that in ATRA-sensitive pancreatic cancer cell lines, increased expression of CRABP2 as well as the tumor suppressors charged multivesicular body protein 1A (Chmp1A) and p53 occurs after ATRA treatment, whereas Chmp1A knockdown abrogates ATRA-mediated growth inhibition [71]. This suggests that ATRA-induced growth inhibition in human pancreatic cancer cells is mediated, at least in part, via Chmp1A.

Moreover, it was found that the kinesin-related protein HsEg5, which plays an important role in spindle assembly and spindle function during mitosis, also appears to be involved in the ATRA-mediated antitumor effect [72]. ATRA has been shown to inhibit HsEg5 expression in several PDAC cell lines, resulting in the obstruction of progression through the G2/M phase of the cell cycle [73]. This indicates that ATRA inhibits cell growth of pancreatic cancer cells by affecting the bipolar spindle apparatus during mitosis. Furthermore, in some pancreatic cancer cell lines, G0/G1 arrest also appears to be induced by retinoic acid [58,74].

In addition to the evaluation of a combination treatment of retinoids and the standard chemotherapeutic agents for the treatment of PDAC, the combined treatment of retinoids and vitamin D or vitamin D analogues has also been investigated. For example, an early investigation of the combination of ATRA as well as 9-cis-retinoic acid with vitamin D analogues showed increased inhibition (90 %) of PDAC cell lines [47]. In a more recent study, cotreatment of 13-cis-retinoic acid with vitamin D inhibited PDAC cell invasion in vitro [75].

### 2.2 Vitamin C

#### 2.2.1 Metabolism and function

Vitamin C (ascorbic acid, ascorbate) is a micronutrient essential for humans, primates, and guinea pigs that is synthesized by other mammalian species from glucose and galactose [110]. It is easily oxidized to L-dehydroascorbic acid, in which the unsaturated 2,3-dihydroxy group is replaced by a saturated 2,3-diketone function. L-dehydroascorbic acid can be reduced back to ascorbic acid. Vitamin C is highly water soluble, and in solution can be oxidized by atmospheric oxygen to give an equilibrium mixture of ascorbic and dehydroascorbic acid. Vitamin C has important antioxidant properties and protects cells against oxidative stress [111]. Because of this general cytoprotective role, its importance has been investigated in a variety of clinical conditions including cancer, vascular disease, and cataracts [112].

Major food sources of vitamin C are plants such as citrus fruits, soft fruits, and green vegetables [113]. Ascorbic acid is a permitted antioxidant additive in food, with no specified limits on the level of use [114]. Approximately 70–90 % of vitamin C is absorbed at moderate intakes of 30–180 mg/day [115]. However, at doses above 1 g/day, absorption falls to less than 50 % and absorbed, unmetabolized ascorbic acid is excreted in the urine. Results from pharmacokinetic studies indicate that oral doses of 1.25 g/day ascorbic acid produce mean peak plasma concentrations of 135 μmol/L, which are about two times higher than those produced by consuming 200–300 mg/day ascorbic acid from vitamin C-rich foods [116]. Comparing plasma vitamin C concentrations of 1.25 g administered orally to intravenously, for an oral dose the mean peak plasma concentration was 134.8 ± 20.6 μmol/L, while for intravenous administration, it was 885 ± 201.2 μmol/L [117]. Pharmacokinetic modelling for the maximum tolerated oral dose of 3 g every 4 h predicted peak plasma vitamin C concentrations of 220 μmol/L, while a 50 g intravenous dose resulted in 13,400 μmol/L. The predicted peak urine concentrations from intravenous administration were 140 times higher than those from maximum oral doses.

Vitamin C is a strong antioxidant that can protect against DNA damage caused by free radicals [111]. Oxidative stress, which results from an imbalance between the production of free radicals and the body's ability to counteract their harmful effects [118,119], is implicated in the pathogenesis of acute and chronic pancreatitis. It is well known that vitamin C, which is known for its antioxidant properties, plays an important role in neutralizing harmful reactive oxygen species (ROS) that can potentially damage DNA. Various biological processes, such as peroxisomes, radiation, and mitochondrial activities, produce ROS. Upon intake, vitamin C comes equipped with additional electrons that it can donate to these ROS. The end result of this chemical reaction is the reduction of ROS, thereby eliminating their harmful potential [120].

#### 2.2.2 Vitamin C and pancreatic cancer risk

The protective role of vitamin C especially, the correlation between oral vitamin C intake and the risk of pancreatic cancer has been explored in several observational studies. The EPIC-Norfolk study showed a strong inverse association between serum vitamin C and pancreatic cancer during 17 years of follow-up [121] However, a more recent case-control study nested in the whole EPIC study contradicted these findings [41]. Randomized controlled trials examining the effect of vitamin C supplementation on pancreatic cancer are also limited [122]. A Cochrane systematic review with 20 randomized trials (211,818 participants), assessing vitamin C among other antioxidants reported a null effect, but only one trial of vitamin C supplementation (combined with vitamin E, beta-carotene, and selenium) and pancreatic cancer (ICD-10C25) was included [123]. The randomized Physicians’ Health Study II showed a non-significant 14 % reduction in pancreatic cancer death associated with vitamin C supplementation after a mean follow-up duration of eight years [124]. A meta-analysis by Fan et al., including 17 studies with 4827 pancreatic cancer cases, suggested that the highest vitamin C intake versus the lowest was significantly associated with reduced risk of pancreatic cancer in a pooled group [125]. The detailed results also confirmed this significant correlation and this association remained significant in Caucasian, Asian as well as in a mixed population. A meta-analysis conducted by Chen et al. [126] examining 18 relevant studies assessing the link between antioxidant intake and pancreatic cancer risk, found that a higher dietary intake of vitamin C was significantly associated with a reduced risk of pancreatic cancer. Another meta-analysis of 20 observational studies comprising nearly 5000 cases of pancreatic cancer showed a significant inverse association between vitamin C intake and risk of pancreatic cancer in case-control studies but not in prospective cohort studies. Thus, there is insufficient evidence to conclude a clear relationship between vitamin C intake and risk of pancreatic cancer [122]. A recent umbrella review, based on the existing systematic reviews and meta-analyses used 22 cancer outcomes within 3562 articles and confirmed that vitamin C intake was related to lower incidence of pancreatic cancer and total cancer occurrence [127]. While some studies suggest a potential protective effect of vitamin C against pancreatic cancer, the evidence is not consistent across all studies and study types. It is important to note that these studies have limitations including potential methodological flaws, and the results may be affected by confounders like lifestyle patterns and biases including recall and selection [122].

#### 2.2.3 Vitamin C and pancreatic cancer

Vitamin C in pharmacological concentrations in the millimolar range has been studied for several years regarding its tumor cell-specific cytotoxic effect. Böttger et al. delivered a comprehensive overview of high-dose vitamin C in different cancer entities [128]. As for many other tumor entities, there are numerous reports on the efficacy of high-dose ascorbate in inhibiting growth of tumor cells and killing them in vitro and in animal models for PDAC (Table 1) [81,87,129]. For example, in a PDAC mouse model, treatment with 4 g/kg daily for 45 days alone resulted in an almost 50 % reduction in tumor mass [92]. Regarding the therapeutic benefits of ascorbate, its effectiveness was investigated in preclinical studies not only as a monotherapy, but also in combination with common methods of tumor treatment. Several publications have shown that high-dose vitamin C can significantly increase the effectiveness of therapy with ionizing radiation, both in vitro (4 mM ascorbate) and in vivo (4 g/kg/d) [89,130] and on the other hand minimizes the damage to healthy tissue caused by radiotherapy [131]. Promising results have also been generated regarding the combination of pharmacological vitamin C concentrations with standard chemotherapeutics. In several preclinical studies, the combination of vitamin C with gemcitabine led to synergistic cytotoxicity and growth inhibition, even in gemcitabine-resistant PDAC cell lines [91,132]. For example, the combination with ascorbate in the PDAC mouse model reduced the necessary gemcitabine dose and achieved a 50 % reduction in tumor growth in gemcitabine-resistant tumors [91]. In particular, the authors attributed the synergistic effects to ascorbate-induced hydrogen peroxide formation in combination with gemcitabine-mediated genome instability. The combination treatment of high-dose ascorbate and FOLFIRINOX also showed a significant increase in efficacy in the PDAC mouse model compared to FOLFIRINOX monotherapy, by almost doubling the survival rate after 28 days of treatment [88].

The clinical use of vitamin C in pancreatic cancer treatment is under investigation since 2009 (NCT00954525) and is still the object in several clinical trials (Table 2). Actually, a total of 18 clinical trials on the efficacy of pharmacological ascorbate in pancreatic cancer have been registered in an online database via the website ClinicalTrials.gov [133]. Of these, three have been withdrawn (NCT03908333, NCT03797443, NCT03697239), seven have been completed, three have been terminated, and five are currently active or recruiting (NCT01852890, NCT04150042, NCT03541486, NCT04033107, NCT02905578). The trials include seven phase I (NCT02896907, NCT01654861, NCT01852890, NCT04150042, NCT00954525, NCT01364805, NCT01049880), four phase I/II (NCT03908333, NCT03797443, NCT03410030, NCT03697239), and seven phase II studies (NCT03541486, NCT04033107, NCT01515046, NCT03146962, NCT01555489, NCT01905150, NCT02905578). In the completed studies, very good tolerability of high-dose vitamin C was consistently demonstrated, with a tendency to increased OS with combination treatment compared to standard therapy [86,92,131,134]. While vitamin C's potential as a therapeutic agent in pancreatic cancer is promising, it is important to note that many of these studies are in early stages, and of small populations (less than 100 participating patients). Obviously, phase III clinical trials are urgently needed.

#### 2.2.4 Antitumor mechanism of vitamin C

The pro-oxidant mechanisms of how vitamin C potentially affects cancer cells are largely explored [81,135,136]. In pancreatic cancer cells, in accordance with numerous other tumor entities, it was confirmed that treatment with ascorbate induces hydrogen peroxide (H_2_O_2_) formation in a concentration- and time-dependent manner [87]. Millimolar ascorbate concentrations induce the formation of hydrogen peroxide extracellularly, whereupon it diffuses into the cells. In the presence of catalytic metal ions, especially Fe^2+^, the Fenton reaction occurs intracellularly, in which hydrogen peroxide is converted to the highly reactive hydroxyl radical, which in turn can damage cellular structures such as DNA, proteins, and lipids. While healthy cells are protected from ROS by various protective mechanisms such as the enzyme catalase or glutathione, tumor cells often have reduced antioxidant protection systems. This, as well as the significantly increased concentrations of free iron in tumor cells are two possible explanations for the tumor cell-specific effect of high-dose ascorbate [128]. Furthermore, it could be shown that the sensitization of PDAC cells to radiation treatment by ascorbate is based on hydrogen peroxide formation [137,138]. In addition, the synergistic effect of high-dose ascorbate and DNA methyltransferase inhibitors in vitro and in the PDAC mouse model could be attributed to an increased pro-oxidative effect [139].

In pancreatic cancer cells, it was shown that ascorbate-induced cell death occurs caspase-independently and is accompanied by the formation of autophagosomes. Similarly, autophagy inhibitors were able to suppress ascorbate-induced cell death [80]. More recent studies have shown additional mechanisms such as epigenome regulation, oxygen sensing, immunomodulatory functions, inhibition of EMT as well as kinase activity regulation [82,83,87,111,128,140,141]. For example, a modulation of hypoxia inducible factor 1 subunit alpha activity by high-dose ascorbate has been demonstrated in pancreatic cancer, as well as a more than 50 % reduction in the expression of the vascular endothelial growth factor, both in vitro and in vivo [85]. Furthermore, ascorbate led to tumor cell-specific NAD^+^ depletion in PDAC cells, resulting in ATP depletion and ultimately cell death. In addition, increased α-tubulin acetylation was detected, which reduced motility and mitosis [92]. Recent data suggests that high-dose vitamin C treatment promotes pancreatic cancer cell death by inhibiting glucose metabolism leading to a disruption of mitochondrial function [84]. Furthermore, vitamin C was observed to inhibit the Wnt/β-catenin signaling pathway and Snail-mediated EMT marker expression, further suppressing metastasis in vivo [84,92,129].

In summary, while the exact mechanisms are not fully understood, vitamin C might affect pancreatic cancer risk through its antioxidant properties and supposedly inhibits cancer cell growth and promotes cell death, at least in part by disrupting cancer cell metabolism and cancer spread.

### 2.3 Vitamin D

#### 2.3.1 Metabolism and function

Vitamin D in its initial form, the unhydroxylated state (cholecalciferol), is formed in the skin from 7-dehydrocholesterol. This requires the absorption of ultraviolet B photons, which is why exposure to solar radiation plays an important role in endogenous vitamin D synthesis [142]. In addition, vitamin D intake through the diet of certain foods, such as fatty sea fish in particular, is considered to be of minor importance [143]. To perform its physiological functions, cholecalciferol must be converted into its biologically active form. In a first step, it is bound to the vitamin D binding protein (VDBP) in the blood and transported to the liver, where it is converted to 25-hydroxyvitamin D (25(OH)D). Predominantly in the kidneys, but also partially in target tissues, 25(OH)D is converted by further hydroxylation to 1,25-dihydroxyvitamin D (1,25(OH)_2_D, or calcitriol) [144].

Calcitriol mediates its action by binding to the nuclear vitamin D receptor (VDR) in target cells, which after calcitriol binding forms a heterodimer with RXR and induces gene transcription by binding to the promoter region of certain target genes [145]. Vitamin D-induced DNA regions include a total of about 1000 genes encoding a number of proteins that are particularly involved in calcium and phosphate homeostasis, stimulating, for example, intestinal absorption of both substances and calcium resorption from bone. While initially vitamin D was thought to have an exclusively endocrine function related to calcium homeostasis, the observation of the expression of both the hydroxylase for calcitriol formation and the VDR in a variety of cells (for example, gut epithelia, immune cells, and cancer cells) throughout the body suggested that calcitriol may have additional autocrine and paracrine effects [[146], [147], [148]].

The use of high-dose calcitriol in tumor diseases is viewed critically due to the associated risk of hypercalcemia and hyperphosphatemia [149]. For this reason, various analogues of vitamin D have been developed, which possess its para- and autocrine effects, but without developing endocrine effects and the associated side effects [142].

Regarding the optimal vitamin D plasma concentrations, different institutions provide different information. In the U.S., serum levels of 25(OH)D are classified by the Food and Nutrition Board of the Institute of Medicine as follows: deficiency, <30 nM (12 ng/mL); inadequacy, 30–50 nM (12–20 ng/mL); normal, 50–125 nM (20–50 ng/mL); high, >125 nM (50 ng/mL) [150].

#### 2.3.2 Vitamin D and pancreatic cancer risk and incidence

Besides the antitumor efficacy of vitamin D and its analogues, which is mainly reported from in vitro and animal models, a possible influence of vitamin D on cancer risk and prognosis of tumor patients is also discussed. However, studies are partly contradictory and there is a lack of randomized controlled trials to clearly establish a relationship [19]. Epidemiological studies have not yet provided a clear picture to answer this question. In general, the incidence of pancreatic neoplasms is three to four times higher in northern latitudes than in areas near the equator [151]. Concordantly, with few exceptions, the incidence for pancreatic tumors is also higher in countries with low UVB irradiation [152]. Furthermore, prospective studies have shown that high vitamin D intake is inversely correlated with the risk of developing pancreatic tumors [153]. In addition, certain SNPs within the genes of proteins involved in vitamin D metabolism (VDR, VDBP, and hydroxylases) have been shown to correlate with pancreatic cancer risk [19]. With regard to pancreatic cancer treatment, it has been observed that deficiency of serum vitamin D prior to treatment is associated with increased inflammatory biomarkers and shorter OS [154].

However, it should be mentioned that opposite correlations have also been observed. For example, a meta-analysis of observational studies found no significant association between vitamin D intake and the risk of pancreatic tumors [155]. Another pooled analysis even concluded that elevated dietary vitamin D intake was associated with an increased risk of developing pancreatic cancer [156]. Thus, large-scale RCT studies are needed to address the question of a possible association of vitamin D intake or plasma vitamin D levels with the development of pancreatic tumors.

#### 2.3.3 Vitamin D and pancreatic cancer

Vitamin D has been shown to play an important role in cell growth, differentiation, apoptosis, and angiogenesis, all factors that are crucial for tumor initiation and progression [157]. Numerous studies on the antitumor effect of vitamin D have been performed on breast, colon, and prostate cancer cell lines and have yielded promising results, as summarized by Barreto et al. [142]. Due to the calcemic activity of vitamin D, which leads to side effects when used in high doses over a long-term period, vitamin D analogues have increasingly become the focus of research. These noncalcemic analogues exhibit antitumor effects through activation of the VDR while at the same time reducing side effects due to their lower activity in stimulating intestinal calcium absorption and bone resorption compared to calcitriol [158]. However, there are also first publications for vitamin D and especially vitamin D analogues regarding the antitumor effect against pancreatic cancer (Table 1). For example, an inhibition of cell proliferation and cell growth by induction of cell cycle arrest in pancreatic cancer cells was found for calcitriol analogues. Furthermore, induction of apoptosis, reduced migration and invasion of pancreatic cancer cell lines, cells from tumor tissues, and pancreatic xenografts in mice were observed [47,49,105,107,[159], [160], [161]]. For example, inhibition of EMT by the calcitriol analog MART-10 has been demonstrated in pancreatic cancer cells [100]. The vitamin D analogue calcipotriol was able to increase the efficacy of oncolytic viroimmunotherapy in a PDAC mouse model by attenuating fibrosis in the pancreatic stroma, thereby increasing immune cell infiltration into the tumor environment and viral delivery to the tumor [95]. The same mechanism could also increase the efficacy of common chemotherapeutics for the treatment of PDAC. Calcipotriol has also been shown to increase the efficacy of gemcitabine treatment when both compounds were combined [101], similarly an increased response to chemoradiotherapy in pancreatic cancer patients was shown when calcipotriol was added to the treatment [96].

Based on the promising in vitro and animal studies on the antitumor efficacy of vitamin D, attempts have been made to substantiate these results in clinical trials (Table 2). A first phase II clinical trial was conducted in 2002 by Evans et al. with the vitamin D analog seocalcitol in patients with advanced (unresectable) pancreatic cancer [104]. While this study found good tolerability of the compound, no antitumor efficacy was recorded. In a phase II clinical trial, the efficacy of a combination therapy of calcitriol and docetaxel was investigated in patients with non-resectable, incurable pancreatic cancer [162]. While a moderate deceleration of tumor progression was observed compared to historical results with docetaxel alone, the efficacy did not suggest superiority of this form of therapy over standard gemcitabine treatment. A pilot study with the vitamin D analogue paricalcitol in combination with chemotherapy showed good tolerability in patients with pancreatic cancer [108].

To our knowledge, no other results of clinical trials of vitamin D for pancreatic cancer treatment have been published to date. However, several phase I and phase II clinical trials are currently underway, the results of which could provide important insights into the benefits of vitamin D and its analogues in the treatment of pancreatic cancer. In these studies, paricalcitol is the focus. Currently, there are two ongoing phase II trials investigating the efficacy of combining the standard therapy of gemcitabine and nabP with paricalcitol in patients with advanced/metastatic PDAC compared to treatment without paricalcitol (NCT03520790 and NCT04617067). Theoretically, the addition of vitamin D or its analogues could help loosen the dense tumor stroma that is typical of PDAC, making it easier for standard chemotherapeutic drugs to reach the tumor and potentially increasing treatment success.

In addition, a clinical investigation of the effect of neoadjuvant paricalcitol treatment on the microenvironment of pancreatic tumors prior to surgery is ongoing (NCT02030860). Here, initial evidence may be gathered as to whether the stromal changes observed in animal models due to vitamin D also increase the response to various forms of therapy in humans. Another phase I study is currently evaluating the combination of liposomal irinotecan, 5-fluorouracil, leucovorin, and various concentrations of paricalcitol as second line therapy in PDAC patients (NCT03883919). Most recently, a phase II trial evaluating the efficacy of combining the PD1 inhibitor pembrolizumab with paricalcitol in PDAC was completed (NCT03331562).

#### 2.3.4 Antitumor mechanism of vitamin D in pancreatic cancer

The specific antitumor mechanism of vitamin D is currently not fully understood, but can most likely be predominantly attributed to the altered expression of specific gene segments regulated by the VDR [19]. Via activation of the VDR, vitamin D has been shown to regulate the activity of over 60 genes, affecting cell differentiation, proliferation, metastasis, angiogenesis, as well as cell cycle [163]. For example, vitamin D has been found to inhibit cell proliferation of pancreatic cancer cells, among others, by increasing the activity of the cyclin-dependent kinase inhibitors p21, p25, and p27, leading to cell cycle arrest [97,[164], [165], [166]]. Furthermore, it was shown that vitamin D as well as its analogues also initiate apoptosis in cancer cells by upregulating the expression of p21 as well as the tumor suppressor gene p53 [164]. In addition, the authors were able to show that vitamin D upregulates the proapoptotic factor bcl-2-like protein 4, while downregulating the expression of the anti-apoptotic factor B-cell lymphoma 2 in glioma cells. A signaling pathway that also seems to be particularly important in gastrointestinal malignancies is the hedgehog pathway, whose activity is also increased in pancreatic tumors [167,168]. In vitro, vitamin D has been shown to act as a hedgehog pathway inhibitor, thereby inhibiting the growth of pancreatic cancer cell lines by around 75 % [98]. Furthermore, there is evidence that vitamin D can influence tumor growth as well as tumor-induced angiogenesis. Bernardi et al. showed that vitamin D decreases the expression of the angiogenic factor angiopoietin 2 in different tumor cell lines and inhibits the proliferation of tumor-derived endothelial cells, which play an important role in the attachment of a tumor to the vascular system [169]. Other molecular factors that play a role in both initiation and progression of pancreatic tumors, such as Wnt signaling and the activity of the transcription factor Forkhead box M1 could also be influenced in individual studies, thereby reducing tumor growth in vitro [102,170]. Moreover, in pancreatic cancer cells, vitamin D has been shown to inhibit the formation of interleukin 8, which plays a crucial role in tumor-induced angiogenesis and whose formation is often increased in pancreatic cancer cells [171].

Another factor that complicates the treatment of PDAC is the so-called stromal density of the tumor and its environment. More specifically, the PDAC stroma is densely fibrotic, which complicates the delivery of chemotherapeutic agents as well as the recruitment of immune cells into the tumor environment. For this reason, the possibility of modulating this stroma to increase the effectiveness of therapies such as viroimmunotherapy is being investigated [172]. In this context, it has been shown that vitamin D and its analogues can lead to reprogramming of the pancreatic stroma, thereby reducing fibrosis and equally enhancing drug delivery of chemotherapeutic agents and the efficacy of viroimmunotherapy in PDAC [95,101]. Another important factor limiting the efficacy of chemotherapeutic agents in conventional cancer therapy is the expression of efflux proteins in cancer cells, which reduce the efficacy of treatment and cause resistance to therapy [93]. Interestingly, Gilzad-Kohan et al. demonstrated in pancreatic cancer cells that calcitriol can increase the uptake of gemcitabine into tumor cells by down-regulating the expression of the gemcitabine efflux proteins multidrug resistance protein 1 and 5 [94]. Accordingly, pretreatment with calcitriol prior to gemcitabine treatment resulted in an increase in cell death, which was also confirmed in other studies in vitro and in mice [173].

Although other mechanisms explain the antitumor efficacy of vitamin D than those observed for vitamin C, there is also evidence for an influence of vitamin D on iron homeostasis and the induction of oxidative stress. Bajbouj et al. were able to show in breast cancer cell lines that vitamin D influences the expression of various iron regulatory genes, induces iron depletion in the cells, and shifts the cellular redox balance towards oxidative stress [174]. This ultimately led to reduced cell survival after treatment with vitamin D.

## 3 Discussion

Vitamins A, C, and D are essential nutrients for the human organism. Among other things, vitamin C acts as a protective substance that prevents damage to cellular components. On the other hand, both vitamin A and vitamin D play an important role in cellular growth processes and can regulate the expression of various genes through their transcription-influencing effect. Although the role of these three substances in the development of tumor diseases is largely unknown, these functions at least make a certain connection conceivable and lay the foundation for the possible use of these vitamins in the treatment of tumor diseases, such as PDAC.

While current tumor therapeutics and most novel therapeutic approaches are aimed directly at damaging the tumor cells, this approach is problematic in the case of PDAC. Due to the activity of PSCs, which ensure the formation of a connective tissue-like structure, the stroma, in the tumor environment, it is difficult for tumor therapeutics to reach the tumor cells and exert their effect at the target site [175]. The therapeutic application of vitamin D or its synthetic analogues seems particularly promising here. If the stroma-modulating effect of these compounds can also be confirmed in the ongoing clinical trials, this would provide a significantly larger target area for other tumor therapeutics, which could considerably increase their effectiveness. This aspect makes vitamin D particularly interesting for use in PDAC therapy. Especially in combination with the immunomodulatory effects of vitamin D, a significant increase in the effectiveness of viroimmunotherapy or other immune-based therapeutic approaches would be conceivable, which has not yet been successfully investigated in PDAC. In addition, it has recently been reported that vitamin D reduces the risk of colitis induced by immune checkpoint inhibitors [176].

However, one problem with the therapeutic use of vitamin D and its analogues could be that reduced VDR expression in tumor tissue correlates with low differentiation, tumor progression, and low survival time [177], so that the use of vitamin D could be more difficult, especially in the patient population with the lowest life expectancy. It is important to await the results of the ongoing human studies to be able to draw conclusions about the potential success of supplementing standard therapy with vitamin D or its analogues.

Regarding the tumor therapeutic use of the various forms of vitamin A, the clinical data situation for pancreatic tumors is very limited. Since ATRA is the major biologically active form of vitamin A [178], there is an urgent need for further clinical studies investigating the efficacy of this vitamin A form in pancreatic cancer to confirm the promising preclinical studies. Similar to compounds with biological calcitriol activity, the stroma-modulating effect seems particularly interesting. Regarding this mode of action, there are also initial indications from a clinical study with ATRA [63]. This point of attack of both vitamins could herald a new approach to the treatment of pancreatic tumors. To optimize this therapeutic approach, the exact mechanisms of the effects of vitamins A and D on PSC and the tumor stroma should be deciphered in future studies to optimize the combination with conventional therapeutic approaches and identify molecular determinants of successful therapy.

A decisive point that must be considered when using vitamins for tumor therapy is the necessity of certain required cellular structures to develop the effect of the respective substance. In the case of vitamins A and D, these are the specific nuclear receptors VDR for vitamin D and RAR or RXR for vitamin A [19,65]. While it has been shown for RAR and RXR that these receptors are frequently downregulated in PDAC tissue compared to normal pancreatic tissue [179], it was shown that pancreatic cancer cells frequently overexpress VDR and that VDR is also present in PSC, which makes these cells potentially accessible for treatment with vitamin D [101,106]. In individual cases, gene screening of a tumor biopsy could provide information on receptor expression and indicate whether supplementary therapy with vitamin D or vitamin A is advisable.

In the case of high-dose ascorbate, however, the antitumor mechanism of action appears to be based on completely different principles according to current knowledge. In contrast to vitamins A and D, ascorbate is currently not known to directly influence gene expression through receptor activation. Here, proteins that are associated with iron metabolism, the redox balance, hydrogen peroxide and vitamin C uptake are of primary importance, as summarized by Leischner et al. [180]. According to current research, these mechanisms appear to be involved in the main cytotoxic mechanism of action of pharmacological vitamin C concentrations against tumor cells.

Of particular interest in this context are aquaporins (AQP), which are most likely responsible for cellular hydrogen peroxide uptake after extracellular formation. In addition, various PDAC cell lines have been shown to overexpress different AQPs compared to healthy pancreatic tissue [181,182]. AQP3, but also AQP5 and AQP8 appear to be of interest here. There are initial indications that both vitamin C and gemcitabine can increase the expression of certain AQPs in tumor cells [183,184], which could explain the synergistic toxicity of both substances towards PDAC cells observed in vitro and in animal experiments. Iron metabolism proteins are also thought to be essential for the cytotoxicity of high-dose vitamin C, which are responsible for the frequently increased labile iron pool in tumor cells. Proteins for iron import, such as the transferrin receptor 1 and the divalent metal transporter 1, but also proteins for iron export, such as ferroportin, are particularly relevant here. Even though the role of these proteins in ascorbate-induced cytotoxicity in tumor cells is still uncertain, their expression level, in addition to AQP expression, could serve as a molecular marker for predicting the success of high-dose ascorbate treatment in tumor patients.

Limitations are strongly dependent on the respective vitamin. Vitamin A and vitamin D can accumulate and cause all symptoms of a hypervitaminosis A and D, which in turn can also have an influence on the side effects of standard therapy. For this reason, the plasma levels of vitamin A and D should be checked regularly during therapeutic use. High-dose vitamin C can be immediately removed by the kidneys, but requires good kidney function without kidney stones. A possible interaction when combining the vitamins would be, for example, the induction of kidney stones by high doses of vitamin D. Kidney stones represent a relative contraindication for high-dose vitamin C. Interactions with standard therapy are rare for high-dose vitamin C and an additional benefit is described in many smaller studies with combined administration [128]. Nevertheless, the redox activity should certainly be taken into account in individual cases.

Due to the complexity of gene expression, it is sometimes difficult to distinguish between correlation and causality [185]. In the case of the vitamins described, the quality of the individual in vitro and in vivo studies varies greatly and it is not always clear, for example, whether there was a vitamin deficiency and, if so, whether this is the cause or consequence of the disease.

An important aspect when transferring the promising in vitro results to the physiological situation is the vitamin supply of the patients. As the concentration of many vitamins in cell culture is often reduced compared to physiological conditions, this could represent an overestimation of efficacy compared to the situation in adequately supplied test subjects. On the other hand, an undersupply of some nutrients, including vitamin C, is often observed in tumor patients, so that the effectiveness of tumor therapy could depend on the individual supply status of the individual patient.

These aspects, in particular the exact antitumor mechanisms of action of the individual vitamins, should be investigated in future trials. It is particularly important to investigate the promising preclinical results and initial findings from phase I and phase II trials in randomized, controlled phase III trials with a sufficient number of subjects in order to validate a possible benefit over the current standard therapy. This will presumably make it possible to optimize the use of these compounds in tumor therapy regarding an optimal therapy regime and to identify patient groups that particularly benefit from vitamin therapy. Irrespective of the anti-tumor efficacy, the usefulness of a general supplementation of the treatment with vitamins A, C, and D should be considered. Particularly in view of the frequent nutrient deficiency in tumor patients, this would appear to make sense. In addition, regardless of its anti-tumor efficacy, there is strong evidence, particularly for vitamin C, that the use of high doses can reduce the severe side effects of many standard chemotherapeutics [92,186]. This alone could potentially improve the quality of life of many tumor patients.

A possible design for a clinical trial on PDAC patients could be based on the use of high-dose vitamin C as an addition to the respective standard therapy. First, a glucose-6-phosphate dehydrogenase deficiency would have to be ruled out, kidney function checked and the presence of kidney stones excluded. Vitamin C would then be dosed in 15 g increments on a weekly basis to a target dosage of between 1 and 1.5 g/kg body weight (light-protected application and diluted 1 + 1 with isotonic saline solution or another carrier solution, whereby the influence of the chloride ions on kidney function would have to be monitored by regularly checking the kidney function). Administration of vitamin D_3_ would supposedly stop stroma formation to make the PDAC more accessible for chemotherapy as well as high-dose vitamin C. To avoid kidney stones in combination with high-dose vitamin C, the cholecalciferol concentration should be checked at least every two months (target range 40–50 ng/mL) to rule out hypervitaminosis D. These effects could possibly be avoided with the alternative use of vitamin D receptor agonists. If vitamin D_3_ is used, supplementary magnesium citrate could help to reduce formation kidney stone formation. In addition, vitamin A levels should be measured every three months to ensure normal to high levels, but hypervitaminosis A should also be avoided.

In addition to determining the vitamin levels, attention should also be paid to whether findings from molecular genetic examinations of the tumor, which are obtained using next generation sequencing, for example, can have an influence on the necessary dosages of individual vitamins or even represent contraindications. Recent research indicates that gastric cancer is affected by the state of the microbiome and further studies are needed to clarify the role of the microbiota in the establishment and progression of PDAC, which further increases the complexity of this topic [187]. Therefore, artificial intelligence might be a powerful tool that could facilitate the identification of patients that would supposedly have a benefit of additional vitamin treatment and to predict outcomes by evaluating certain blood parameters [188]. Together with other measures, artificial intelligence can also make an effective contribution to developing individualized therapy concepts.

## 4 Conclusion

This review summarized the current state of research on the role of vitamins A, C and D as potential complementary treatment options for PDAC. All three substances influence tumor growth and/or the tumor microenvironment through different, partly overlapping mechanisms. This leads to the assumption of a synergistic efficacy of the vitamins in the treatment of PDAC in combination with the current standard treatment methods.

The next step is to urgently confirm the promising preclinical findings in human clinical trials with PDAC patients. In addition to standard therapy, the administration of high-dose vitamin C in combination with at least adequate vitamin A and D supplementation should be considered in order to demonstrate a possible improvement in the therapy success. Therefore, a decisive contribution could be made to improving the survival time and quality of life of PDAC patients.

## CRediT authorship contribution statement

**Alban Piotrowsky:** Writing – review & editing, Writing – original draft, Methodology, Data curation. **Markus Burkard:** Writing – review & editing, Supervision. **Hendrik Schmieder:** Writing – review & editing, Investigation, Formal analysis, Data curation. **Sascha Venturelli:** Validation, Supervision, Resources, Project administration, Funding acquisition. **Olga Renner:** Writing – original draft, Methodology, Data curation, Conceptualization. **Luigi Marongiu:** Writing – review & editing, Visualization, Data curation.

## Funding

AP, MB, SV, and OR were supported by a grant from the Dr. Hans Fritz Stiftung (funding 3140080501). MB, HS, and SV were supported by a grant from Orthomol pharmazeutische Vertriebs GmbH (funding 3140080701). MB and SV were supported by grants from PASCOE pharmazeutische Praeparate GmbH (grant no. D.31.15100 and D.32.22506). LM and MB were supported by the 10.13039/100022764Ministry of Rural Affairs and Consumer Protection Baden-Wuerttemberg (AZ: 34-9185.90-1).

## Declaration of competing interest

The authors declare the following financial interests/personal relationships which may be considered as potential competing interests:Luigi Marongiu reports financial support was provided by 10.13039/100022764Ministry of Rural Affairs and Consumer Protection Baden-Wuerttemberg. If there are other authors, they declare that they have no known competing financial interests or personal relationships that could have appeared to influence the work reported in this paper.
